# Alleviative effects of α‐lipoic acid on muscle atrophy via the modulation of TNF‐α/JNK and PI3K/AKT pathways in high‐fat diet and streptozotocin‐induced type 2 diabetic rats

**DOI:** 10.1002/fsn3.3227

**Published:** 2023-01-12

**Authors:** Chih‐Yuan Ko, Chi‐Hao Wu, Wen‐Jian Huang, Yangming Martin Lo, Shih‐Xiang Lin, James Swi‐Bea Wu, Wen‐Chung Huang, Szu‐Chuan Shen

**Affiliations:** ^1^ Department of Clinical Nutrition The Second Affiliated Hospital of Fujian Medical University Quanzhou China; ^2^ School of Public Health Fujian Medical University Fuzhou China; ^3^ School of Medical Technology and Engineering Fujian Medical University Fuzhou China; ^4^ Graduate Program of Nutrition Science National Taiwan Normal University Taipei Taiwan; ^5^ Institute for Advanced Study Shenzhen University Shenzhen China; ^6^ Graduate Institute of Food Science and Technology National Taiwan University Taipei Taiwan; ^7^ Graduate Institute of Health Industry Technology Chang Gung University of Science and Technology Taoyuan Taiwan

**Keywords:** α‐lipoic acid, diabetes mellitus, muscle atrophy, proinflammatory cytokines, sarcopenia

## Abstract

Diabetes mellitus (DM) is often accompanied by clinical complications such as sarcopenia. Previous studies have indicated that oxidative stress and insulin resistance (IR) are highly associated with the pathogenesis of diabetic myopathy. α‐lipoic acid (ALA), a potent biological antioxidant, exists abundantly in a variety of plants and vegetables. This study aimed to investigate the ameliorative effect of ALA on muscle atrophy in type 2 diabetic rats induced by high‐fat diet feeding (HFD) plus streptozotocin (STZ) injection. The HFD/STZ‐induced diabetic rats were orally administered 50, 100, or 200 mg/kg body weight ALA once a day for 13 weeks. The results showed that ALA at the tested concentrations significantly increased the soleus muscle mass and muscle fibers in diabetic rats. Proinflammatory cytokines, such as tumor necrosis factor (TNF)‐α, were found to decrease in both the serum and muscle of ALA‐treated diabetic rats. ALA significantly reduced the protein‐expression levels of phosphorylated c‐Jun N‐terminal kinase (pJNK)/JNK, forkhead box O3 (FOXO3), and muscle ring‐finger protein‐1 (Murf1); whereas, it enhanced the protein‐expression levels of phosphoinositide 3‐kinase (PI3K), phosphorylated protein kinase B (pAKT)/AKT, myogenin determination gene D (MyoD), the mechanistic target of rapamycin (mTOR), and myosin heavy chain (MyHC) in the soleus muscle of diabetic rats. The results from this study suggested that ALA treatment may preserve soleus muscle mass, alleviate muscle atrophy by suppressing the TNF‐α/JNK pathway, and ameliorate the PI3K/AKT pathway in HFD/STZ‐induced type 2 diabetic rats.

## Graphical abstract

α‐lipoic acid (ALA) might maintain muscle mass and fibers by enhancing the expression of muscle‐generation‐related proteins, such as MyoD, mTOR, and MyHC, and by reducing the expression of muscle‐degradation‐related proteins, including FOXO and Murf1, thus alleviating muscle atrophy in T2DM rats.

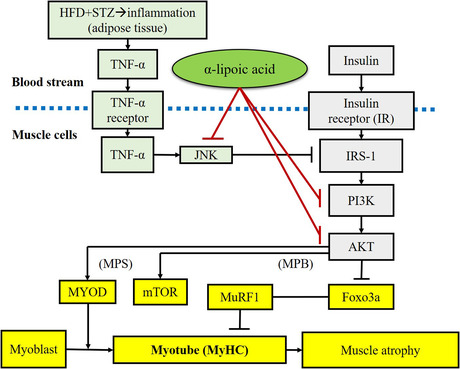

## 1 INTRODUCTION

Diabetes and sarcopenia are major public health problems around the world (Cleasby et al., 2016; Wang et al., 2020). Type 2 diabetes mellitus (T2DM) is mainly attributed to insulin resistance (IR) in the human body, which inhibits effective utilization of carbohydrates, thus causing symptoms of hyperglycemia and hyperinsulinemia. Most patients with T2DM are also diagnosed with conditions such as overweight or obesity, and the severity of these symptoms depends on the degree of IR (American Diabetes Association (ADA), 2020). The risk of T2DM increases with increasing age, metabolic abnormalities, and declining physical activity (American Diabetes Association, 2020). Patients with sarcopenia face similar risks as those with T2DM (Izzo et al., 2021). Loss in muscle mass and strength as well as declining physical functions are very common in patients with T2DM (Cleasby et al., 2016).

Sarcopenic obesity was recently recognized as a disease that confirms the correlation among obesity, IR, and sarcopenia (Cleasby et al., 2016; Izzo et al., 2021; Wang et al., 2020). Persistent chronic inflammation leads to the occurrence of IR, which is considered a major factor in diabetes leading to sarcopenia (Cleasby et al., 2016). IR may inhibit carbohydrate metabolism in muscles, that is, the muscle tissues cannot regulate relevant pathways through insulin, leading to metabolic abnormalities. Glucose‐uptake impairment in the muscles of patients with sarcopenia may also lead to the inhibition of protein production, subsequently resulting in muscle loss (Samuel et al., 2010), due mainly to the effects of insulin on the metabolism and synthesis of proteins in muscles. Protein metabolic synthesis in muscle includes two pathways: muscle atrophy and muscle‐regeneration obstruction. The occurrence of IR also suppresses downstream muscle‐regeneration‐related proteins, such as myogenin determination gene D (MyoD) and the mechanistic target of rapamycin (mTOR). Muscle decomposition was also observed when IR occurred with sarcopenia (Carotenuto et al., 2016).

Moreover, excessive intake of fat may also result in the loss of muscle mass due to lipid accumulation and inflammation in muscle tissues (Kitessa & Abeywardena, 2016). Reactive oxygen species (ROS) produced by high‐fat diet (HFD) lead to a chronic inflammatory response and generate pro‐inflammatory cytokines, such as tumor necrosis factor (TNF)‐α, and interleukin (IL)‐1β or IL‐6 (Kitessa & Abeywardena, 2016). Under normal physiological conditions, an inflammatory response contributes to muscle autophagy and muscle regeneration (Karalaki et al., 2009); however, excessive proinflammatory cytokines could lead to muscle‐cell apoptosis, muscle loss, and eventually muscle atrophy (Carotenuto et al., 2016).

α‐lipoic acid (ALA) is a natural antioxidant abundant in plants and vegetables. Previous studies have demonstrated that ALA exhibits alleviative effects on IR and inflammation, as well as on diabetes and its complications (Ko, Lo, et al., 2021; Ko, Xu, et al., 2021). ALA was reported to preserve skeletal muscle mass in Otsuka Long Evans Tokushima Fatty (OLETF) rats (Hong et al., 2018). Additionally, ALA may contribute to maintaining muscle mass and strength in healthy elderly subjects (Negro et al., 2019). Numerous studies indicate that ALA alleviates oxidative stress and inflammation in skeletal muscle (Hong et al., 2018; Rochette et al., 2015; Rousseau et al., 2016; Shay et al., 2009). Nevertheless, its therapeutic potential and its mechanism in the generation or antidegradation of muscle mass in patients with T2DM are not fully understood. This study aimed to explore the alleviative effect of ALA on muscle atrophy with regard to improving IR and inflammatory response in the muscle tissues of rats with T2DM induced by HFD plus streptozotocin (STZ).

## 2 MATERIALS AND METHODS

### 2.1 Animal experimental procedures

The study was approved by the Institutional Animal Care and Use Committee of National Taiwan Normal University with the certificate number 106042. Eight‐week‐old male Wistar rats were reared in a temperature‐controlled room (22 ± 1°C) under a 12 h light/dark cycle with free access to food and water for the duration of the study. After adapting to the environment for 1 week, rats were fed either a high‐fat diet (HFD) of 60% fat‐derived calories or a normal diet for 4 weeks (Ko, Lo, et al., 2021; Ko, Xu, et al., 2021).

High‐fat diet‐fed rats were intraperitoneally injected with STZ (Sigma, St. Louis, MO) in quantities of 30 mg/kg body weight (BW) to induce diabetes and then fed with HFD for 9 more weeks. A total of 36 rats were randomly divided into 6 groups: the Normal group contained rats fed with a normal diet; the DM group contained diabetic rats fed with HFD alone as the negative control; the Pio group contained diabetic rats fed with HFD and orally administered pioglitazone (Pio, 30 mg/kg BW) daily for 13 weeks as the positive control; and the ALA50, ALA100, and ALA200 groups contained diabetic rats fed daily with HFD and orally administered 50, 100, or 200 mg/kg BW ALA (Sigma) for 13 weeks, respectively (Ko, Lo, et al., 2021; Ko, Xu, et al., 2021). All rats were fasted overnight (8 h) and then sacrificed at the end of the experiment. Blood was collected from the abdominal vein, and the soleus muscle tissues were retrieved when rats were sacrificed.

### 2.2 Blood sample collection

The blood was centrifuged at 12,000 *g* at a temperature of 4°C for 15 min to obtain the serum samples and then was stored at −20°C until analysis.

### 2.3 Biochemical analysis

The levels of glucose, insulin, triglyceride, cholesterol, free fatty acid (FFA), creatine kinase, lactate dehydrogenase, and creatinine of the serum were measured using commercial enzyme‐linked immunosorbent assay (ELISA) kits (Waltham, MA, USA), and the measurements were validated using the standard enzymatic method with an automatic biochemical analyzer.

### 2.4 Measurement of TNF‐α in serum and muscle

The levels of TNF‐α were measured using the commercial kit (Waltham, MA, USA) according to the supplier's protocols.

### 2.5 Histological analysis

Soleus muscle tissue was fixed in a 10% neutral phosphate‐buffered formalin solution and embedded in paraffin. Sections (4–5 μm^2^ thick) were stained with hematoxylin and eosin (HE), and were observed and photographed with the aid of an upright digital imaging microscope.

### 2.6 Tissue protein preparation and measurement

To prepare the muscle protein samples, 0.05 g of the muscle tissue was mixed with 0.5 ml of a lysis buffer and ground eight times (10 s each time). After 1 min of incubation in an ice bath, the homogenized tissue solutions were centrifuged at 20,000 *g* at a temperature of 4°C for 60 min to obtain the tissue protein supernatants. The protein concentration was measured at 595 nm using the Bradford method with a Bio‐Rad protein assay kit (Hercules, CA, USA).

### 2.7 Western blot

Aliquots of the muscle tissue protein samples, each containing 40 μg of protein, were evaluated for the expressions of IRS‐1, PI3K, AKT, pAKT, JNK, pJNK, myoD, mTOR, FOXO3, Murf1, and MyHC. The samples were subjected to 5%, 7.5%, or 10% sodium dodecyl sulfate‐polyacrylamide gel electrophoresis. The separated proteins were electrotransferred to a polyvinylidene difluoride membrane. The membrane was incubated with blocking buffer (phosphate‐buffered saline containing 0.05% Tween‐20 (PBST) and 5% (wt/vol) nonfat dry milk) for 1 h, incubated overnight at 4°C with PBST, and probed with anti‐IRS‐1 (Signalway Antibody), anti‐PI3K, anti‐Akt, anti‐pAKT, anti‐JNK (Signalway Antibody), anti‐pJNK (Signalway Antibody), anti‐MyoD, anti‐mTOR, anti‐FOXO3, anti‐Murf1, anti‐MyHC, anti‐β‐catenin, and anti‐GAPDH, to ensure that a constant amount of protein was loaded into each lane of the gel. The membrane was washed three times (5 min each) in PBST, shaken in a solution of horseradish peroxidase‐conjugated anti‐mouse IgG or anti‐rabbit IgG (Genetex) secondary, washed three times (5 min each) in PBST, and incubated in enhanced chemiluminescence reagent (Millipore). Autoradiography was performed and analyzed using a UVP Biospectrum image system (Level).

### 2.8 Statistical analyses

The data are expressed as mean ± standard error of the mean using SPSS version 22.0 (SPSS Inc.). A one‐way analysis of variance and Duncan's multiple range tests were performed. The significance level was set at *p* < .05.

## 3 RESULTS

### 3.1 Effects of ALA on blood biochemical profiles in T2DM rats

The levels of fasting blood glucose in the DM group were significantly higher than those of the Normal, pioglitazone (Pio), and ALA200 groups (Table 1). In contrast, the level of fasting blood insulin in the DM group was significantly higher than those of the others (Table 1). Additionally, the levels of blood triglyceride and FFA in the DM group were significantly higher than those of the Normal, Pio, ALA50, and ALA200 groups (Table 1); the level of cholesterol in the DM group was significantly higher than those of the Pio, ALA50, and ALA200 groups (Table 1); the blood creatine kinase in the DM group was significantly higher than those of the ALA100 and ALA200 groups (Table 1); and the content of blood creatinine in the DM group was significantly lower than those of the other groups (Table 1).

**TABLE 1:** Profiles of serum biochemistry in rats with type 2 diabetes induced by high‐fat diet and streptozotocin and treated with α‐lipoic acid (ALA) for 13 weeks.

Parameter	Normal	DM	Pio	ALA50	ALA100	ALA200
Fasting blood glucose (mg/dl)	151.2 ± 5.1^c^	428.0 ± 39.0^a^	339.5 ± 104.6^b^	406.2 ± 35.0^ab^	402.3 ± 40.9^ab^	383.5 ± 24.8^b^
Fasting blood insulin (μg/dl)	1.02 ± 0.14^b^	2.31 ± 0.89^a^	0.55 ± 0.03^b^	0.87 ± 0.16^b^	0.69 ± 0.03^b^	0.79 ± 0.06^b^
Total triglyceride (mg/dl)	82.3 ± 34.7^c^	324.5 ± 137.6^a^	123 ± 51.7^c^	167.5 ± 83.3^bc^	248.7 ± 92.5^ab^	95 ± 30.3^c^
Total cholesterol (mg/dl)	83.2 ± 19.8^ab^	116.7 ± 32.8^a^	75.3 ± 11.9^b^	75.7 ± 14.5^b^	99.0 ± 49.8^ab^	77.3 ± 10.6^b^
Free fatty acid (mmol/L)	0.62 ± .018^c^	1.13 ± 0.27^a^	0.81 ± 0.26^bc^	0.88 ± 0.15^b^	0.92 ± 0.10^ab^	0.66 ± 0.18^bc^
Creatine kinase (U/L)	366.0 ± 90.1^ab^	436.7 ± 94.2^a^	311.2 ± 63.3^ab^	338.5 ± 69.7 ^ab^	254.0 ± 92.0^b^	229.3 ± 75.6^b^
Lactate dehydrogenase (mg/dl)	1332.0 ± 602.7^a^	1176.2 ± 388.6^a^	923.7 ± 458.2^a^	1217.8 ± 334.2^a^	936.8 ± 529.1^a^	1085 ± 720.0^a^
Creatinine (mg/dl)	0.534 ± 0.031^a^	0.350 ± 0.026^b^	0.468 ± 0.059^a^	0.468 ± 0.013^a^	0.464 ± 0.099^a^	0.506 ± 0.016^a^

*Note*: DM, rats with diabetes induced by high‐fat diet (HFD; 60% fat) with STZ (30 mg/kg body weight, i.p.); DM + Pio, DM rats gavaged with pioglitazone (30 mg/kg body weight) for 13 weeks; DM + ALA50, DM rats gavaged with ALA (50 mg/kg body weight) for 13 weeks; DM + ALA100, DM rats gavaged with ALA (100 mg/kg body weight) for 13 weeks; DM + ALA200, DM rats gavaged with ALA (200 mg/kg body weight) for 13 weeks; Normal, normal diet. Values were calculated as the mean ± SEM, *n* = 6 for each group. a–c letters indicate significant differences among all samples tested (*p* < .05).

### 3.2 Effects of ALA on anti‐inflammation and fiber length of soleus muscle in T2DM rats

**FIGURE 1:**
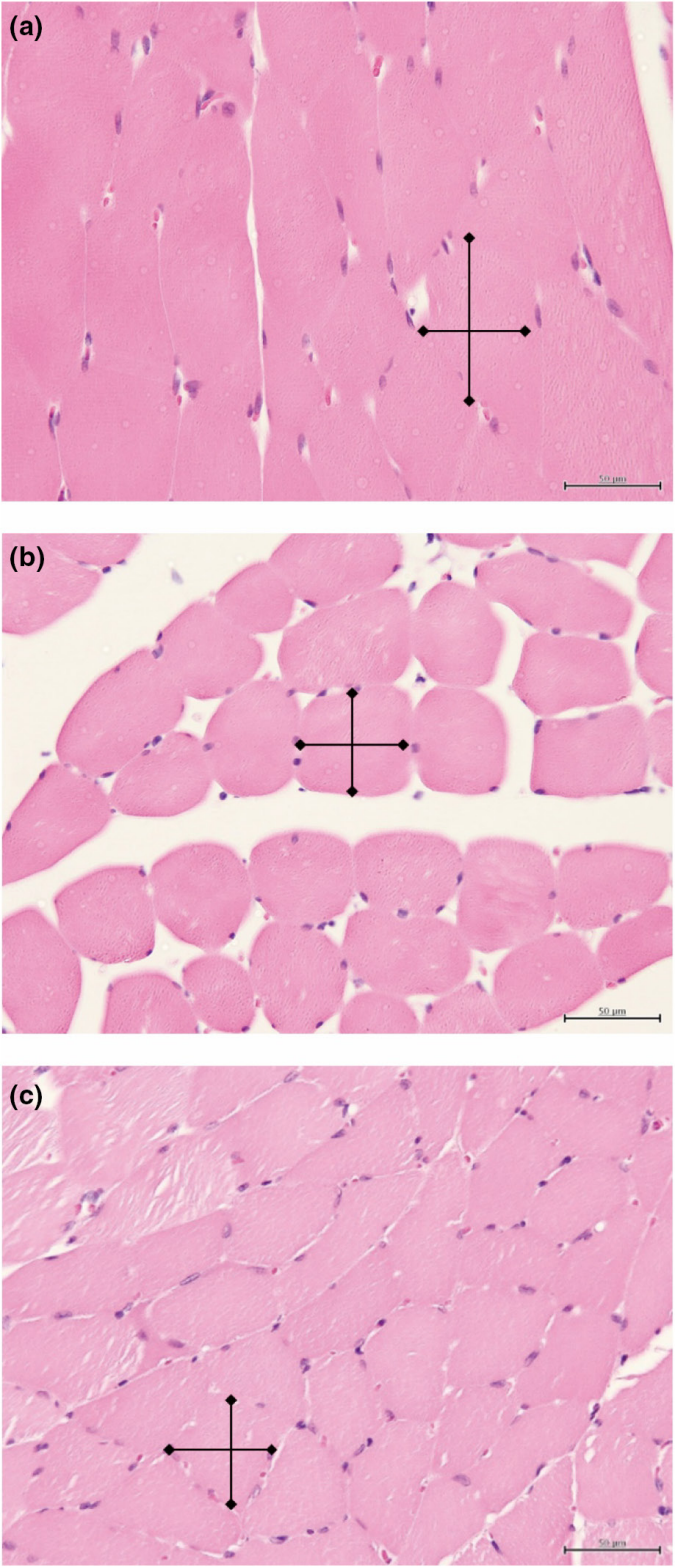
Results of hematoxylin and eosin (H&E) stained soleus muscle sections from Normal (a), DM (b), and (c) DM+ALA200 (×400). Scale bar = 50 μm. Normal, normal diet; DM, rats with diabetes induced by high‐fat diet (HFD; 60% fat) with STZ (30 mg/kg body weight, i.p.); DM+ALA200, DM rats gavaged with α‐lipoic acid (ALA) (200 mg/kg body weight) for 13 weeks.

**FIGURE 2:**
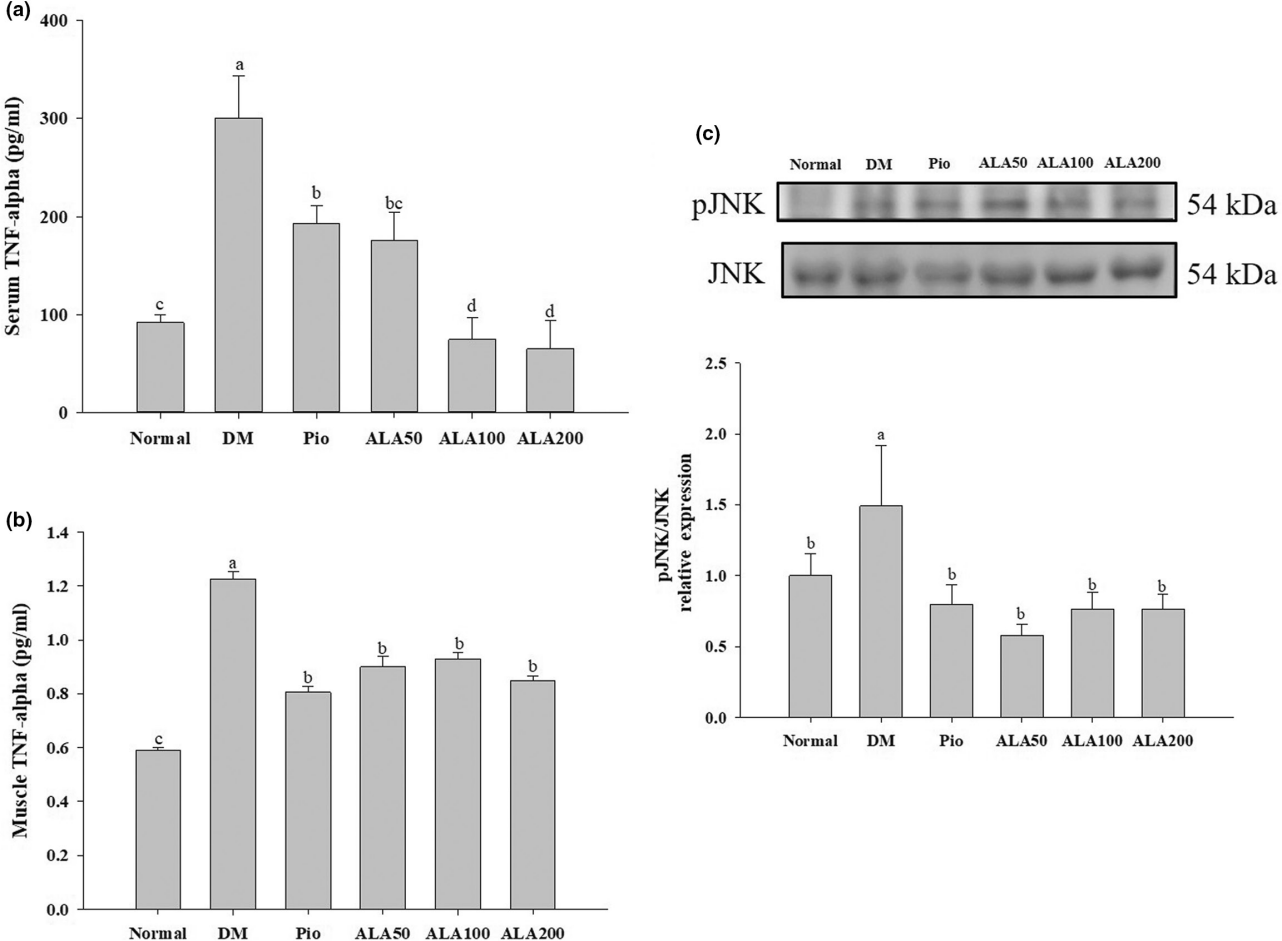
Levels of TNF‐α in serum (a) and muscle (b); protein levels in the inflammatory pathway of pJNK/JNK (c) in rats with type 2 diabetes induced by high‐fat diet and streptozotocin and treated with α‐lipoic acid (ALA) for 13 weeks. Normal, normal diet; DM, rats with diabetes induced by high‐fat diet (HFD; 60% fat) with STZ (30 mg/kg body weight, i.p.); DM + Pio, DM rats gavaged with pioglitazone (30 mg/kg body weight) for 13 weeks; DM + ALA50, DM rats gavaged with ALA (50 mg/kg body weight) for 13 weeks; DM + ALA100, DM rats gavaged with ALA (100 mg/kg body weight) for 13 weeks; DM + ALA200, DM rats gavaged with ALA (200 mg/kg body weight) for 13 weeks. Values were calculated as the mean ± SEM, *n* = 6 for each group. a–d letters indicate significant differences among all samples tested (*p* < .05).

The weight of the soleus muscle in the DM group was significantly lower than those of the Normal and ALA200 groups (Table 2). The relative soleus muscle weight in the DM group was also significantly lower than those of the ALA groups (Table 2), and the soleus muscle fibers in the DM group were significantly shorter than those of the Normal and ALA groups (Table 2). A histological analysis revealed that the muscle fibers in the DM group were significantly shorter than those of the Normal and ALA200 groups (Figure 1). In addition, the TNF‐α levels in both the serum (Figure 2a) and the muscles (Figure 2b) in the DM group were significantly higher than that ofthe other groups. Moreover, the protein expression of phosphorylated c‐Jun N‐terminal kinase (pJNK)/JNK in the DM group was significantly higher than those in the other groups (Figure 2c).

**TABLE 2:** Profiles of soleus muscle in rats with type 2 diabetes induced by high‐fat diet and streptozotocin and treated with α‐lipoic acid (ALA) for 13 weeks.

Parameter	Normal	DM	Pio	ALA50	ALA100	ALA200
Soleus muscle (g)	1.31 ± 0.11^a^	1.01 ± 0.10^c^	1.12 ± 0.09^bc^	1.12 ± 0.10^bc^	1.14 ± 0.13^bc^	1.16 ± 0.13^b^
Relative soleus muscle weight % (g/BW)	0.21 ± 0.01^ab^	0.171 ± 0.02^b^	0.201 ± 0.02^ab^	0.2097 ± 0.02^a^	0.227 ± 0.02^a^	0.224 ± 0.02^a^
Length of soleus muscle fibers (μm)	91.55 ± 9.09^a^	53.18 ± 5.30^d^	56.48 ± 8.59^d^	68.44 ± 6.95^c^	78.28 ± 8.20^b^	79.05 ± 6.45^b^

*Note*: DM, rats with diabetes induced by high‐fat diet (HFD; 60% fat) with STZ (30 mg/kg body weight, i.p.); DM + Pio, DM rats gavaged with pioglitazone (30 mg/kg body weight) for 13 weeks; DM + ALA50, DM rats gavaged with ALA (50 mg/kg body weight) for 13 weeks; DM + ALA100, DM rats gavaged with ALA (100 mg/kg body weight) for 13 weeks; DM + ALA200, DM rats gavaged with ALA (200 mg/kg body weight) for 13 weeks; Normal, normal diet. Values were calculated as the mean ± SEM, *n* = 6 for each group. a–d letters indicate significant differences among all samples tested (*p* < .05).

### 3.3 Effects of ALA on insulin‐signaling‐related protein expression in muscle of T2DM rats

**FIGURE 3:**
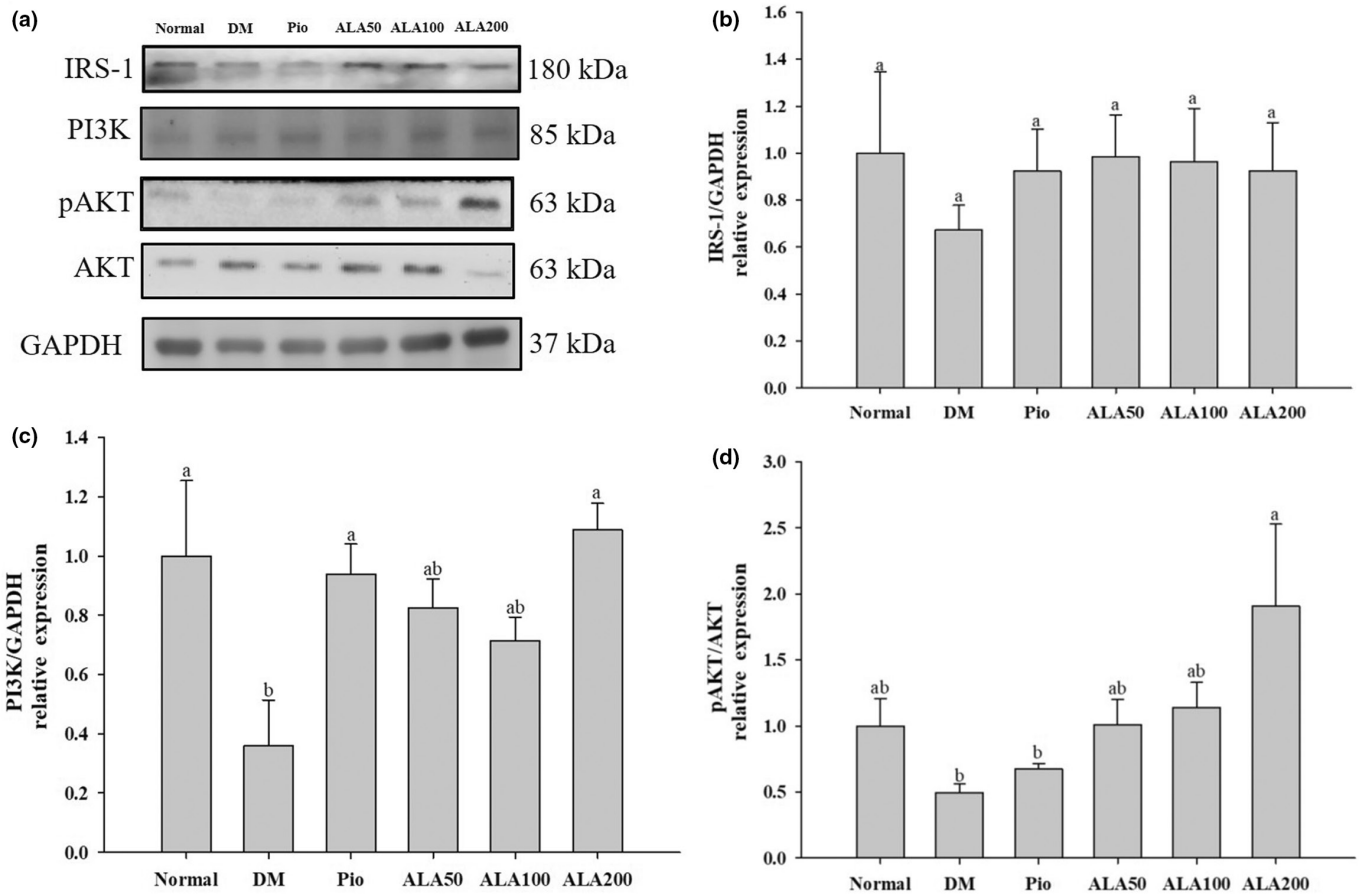
Protein levels of insulin conductive pathways (a) in quantifying relative IRS‐1 (b), PI3K (c), and pAKT/AKT (d) of soleus muscle in rats with type 2 diabetes induced by high‐fat diet and streptozotocin and treated with α‐lipoic acid (ALA) for 13 weeks. Normal, normal diet; DM, rats with diabetes induced by high‐fat diet (HFD; 60% fat) with STZ (30 mg/kg body weight, i.p.); DM + Pio, DM rats gavaged with pioglitazone (30 mg/kg body weight) for 13 weeks; DM + ALA50, DM rats gavaged with ALA (50 mg/kg body weight) for 13 weeks; DM + ALA100, DM rats gavaged with ALA (100 mg/kg body weight) for 13 weeks; DM + ALA200, DM rats gavaged with ALA (200 mg/kg body weight) for 13 weeks. Values were calculated as the mean ± SEM, *n* = 6 for each group. a–b letters indicate significant differences among all samples tested (*p* < .05).

The effect of ALA on insulin‐signaling pathway‐related protein expression is shown in Figure 3a. There was no significant difference in the protein expression of insulin receptor substrate 1 (IRS‐1) among the groups (Figure 3b). The expression level of phosphoinositide 3‐kinase (PI3K) in the DM group was significantly lower than that of the Normal, Pio, and ALA200 groups (Figure 3c). The protein expression of phosphorylated protein kinase B (pAKT)/AKT in the ALA200 group was significantly higher than that of the DM group (Figure 3d).

### 3.4 Effects of ALA on regeneration‐ and decomposition‐related protein expression in muscle of T2DM rats

**FIGURE 4:**
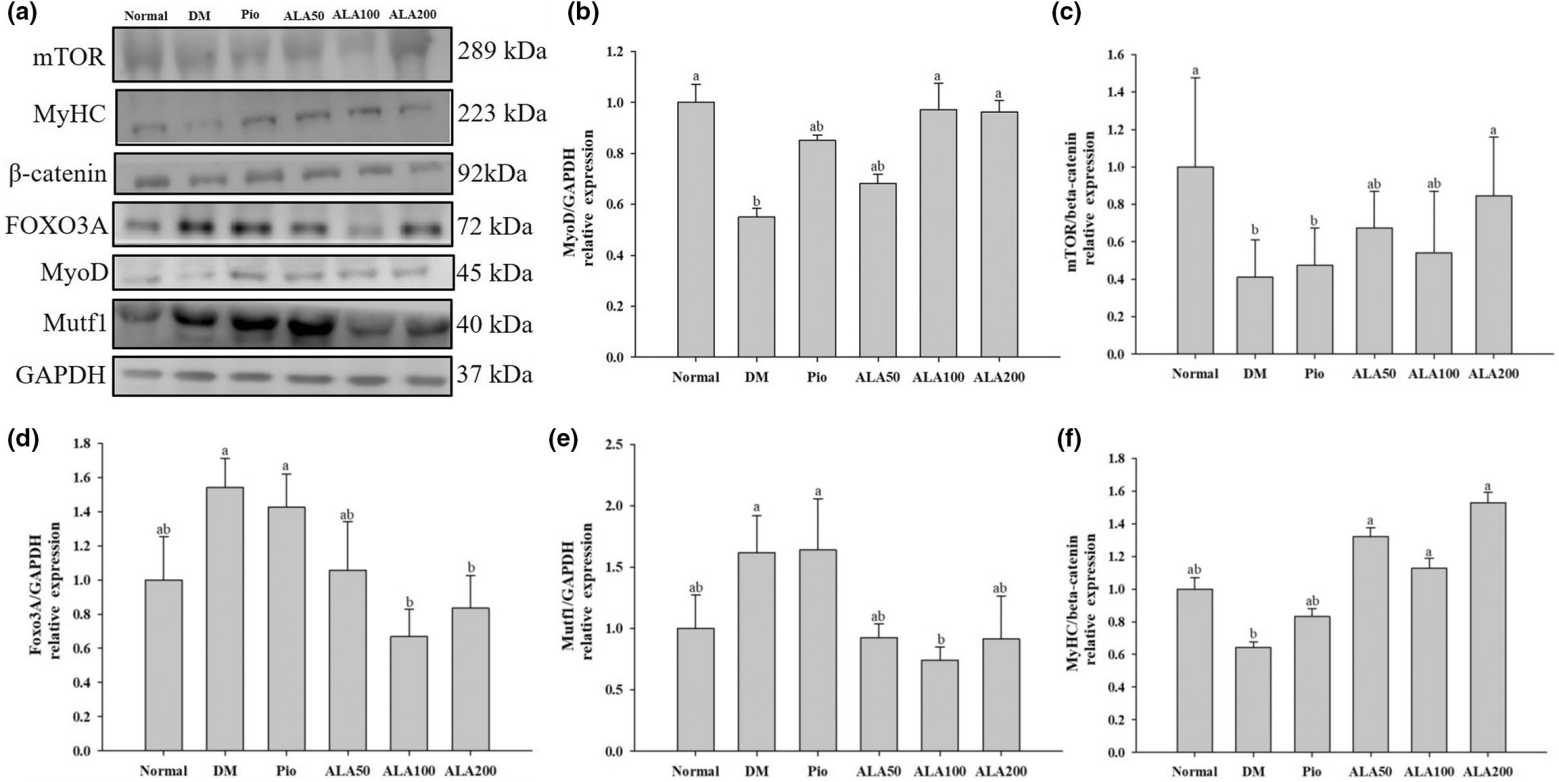
Protein levels of muscle‐regenerative or breakdown pathways (a) in quantifying relative MyoD (b), mTOR (c), FOXO3 (d), Murf1 (e), and MyHC (f) of soleus muscle in rats with type 2 diabetes induced by high‐fat diet and streptozotocin and treated with α‐lipoic acid (ALA) for 13 weeks. Normal, normal diet; DM, rats with diabetes induced by high‐fat diet (HFD; 60% fat) with STZ (30 mg/kg body weight, i.p.); DM + Pio, DM rats gavaged with pioglitazone (30 mg/kg body weight) for 13 weeks; DM + ALA50, DM rats gavaged with ALA (50 mg/kg body weight) for 13 weeks; DM + ALA100, DM rats gavaged with ALA (100 mg/kg body weight) for 13 weeks; DM + ALA200, DM rats gavaged with ALA (200 mg/kg body weight) for 13 weeks. Values were calculated as the mean ± SEM, *n* = 6 for each group. a–b letters indicate significant differences among all samples tested (*p* < .05).

The effect of ALA on the expression of muscle‐regeneration‐ and decomposition‐related proteins is shown in Figure 4a. The protein expression of MyoD in the DM group was significantly lower than those in the Normal, ALA100, and ALA200 groups (Figure 4b). Similarly, the expression level of the mTOR protein in the DM group was significantly lower than those in the Normal and ALA200 groups (Figure 4c). However, the protein expression of forkhead box O3 (FOXO3) in the DM group was significantly higher than those in the ALA100 and ALA200 groups (Figure 4d). The protein expression of muscle ring‐finger protein‐1 (Murf1) in the DM group was significantly higher than that in the ALA100 group (Figure 4e), whereas the protein expression of myosin heavy chain (MyHC) in the DM group was significantly lower than that in the ALA groups (Figure 4f).

## 4 DISCUSSION

**FIGURE 5:**
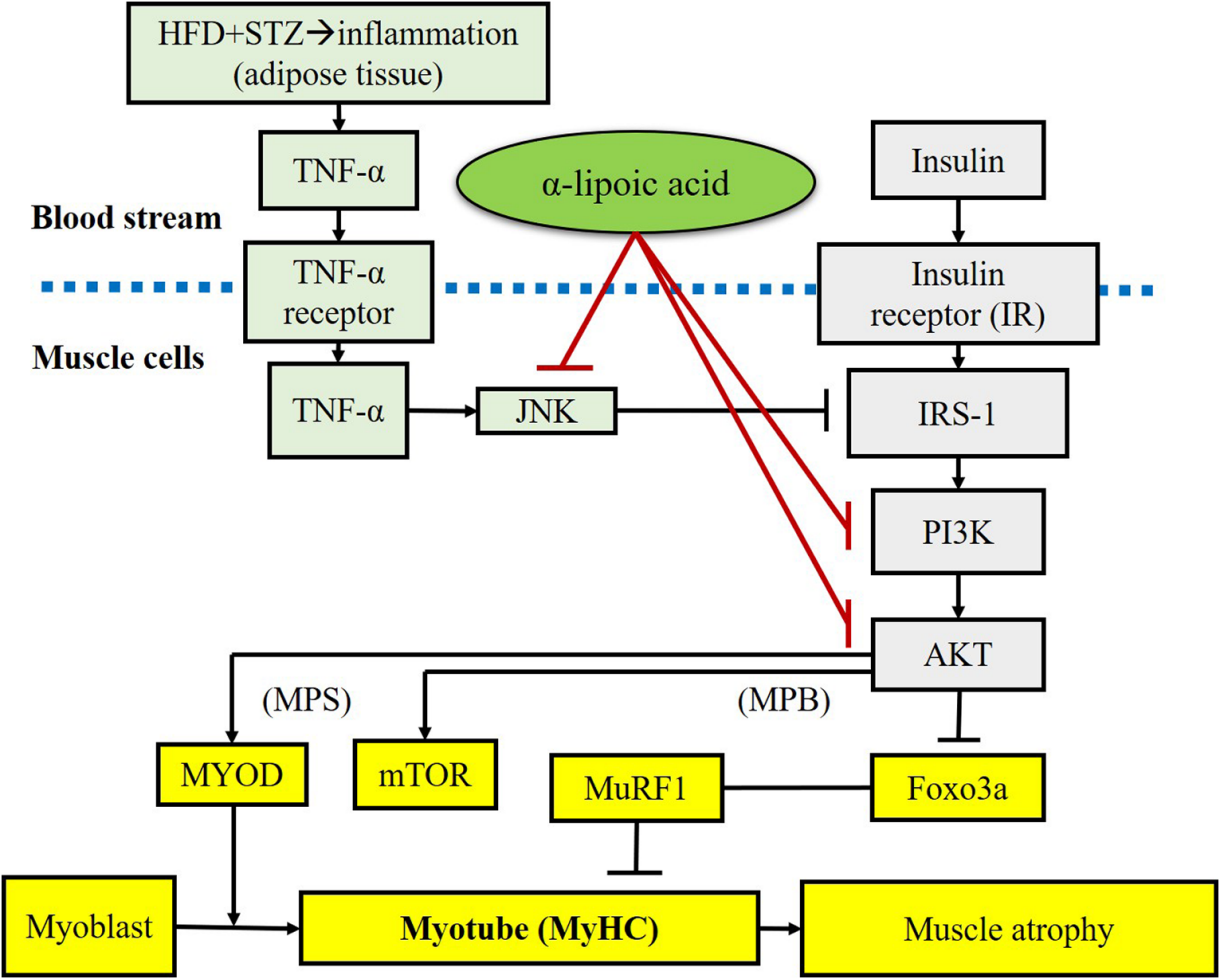
The possible mechanism for α‐lipoic acid alleviation of muscle atrophy in rats with type 2 diabetes induced by high‐fat diet and streptozotocin. MPB, muscle protein breakdown; MPS, muscle protein synthesis.

The present study demonstrates that ALA ameliorated hyperglycemia, hyperinsulinemia, and abnormal lipid profiles were caused by T2DM. In addition, ALA reduced the intensity of inflammatory responses by modulating the TNF‐α/JNK pathway, and it also alleviated IR by regulating the PI3K/AKT pathway in the muscle tissues of rats with HFD/STZ‐induced T2DM. Moreover, ALA might maintain muscle mass and fibers by promoting muscle‐generation‐related protein expression, such as MyoD, mTOR, and MyHC, as well as by reducing muscle‐degradation‐related protein expression, such as FOXO and Murf1, thus alleviating muscle atrophy in T2DM rats (Figure 5).

One study indicated that hyperinsulinemia might be attributed to the occurrence of IR in rats with HFD/STZ‐induced T2DM (Kitessa & Abeywardena, 2016). Excessive lipid‐accumulation‐induced inflammation is a major cause of IR in patients diagnosed with obesity (Tomás et al., 2002). Deemed to be an important cause of obesity, HFD may result in increases in blood triglyceride, cholesterol, and FFA (Ko, Lo, et al., 2021; Liu et al., 2015). Lipid metabolism abnormalities also cause chronic inflammation in the human body. For example, increased serum triglyceride increases inflammation of the arteries in patients with diabetes (Gordillo‐Moscoso et al., 2013). ALA, an excellent antioxidant, was reported to reduce fat oxidation and serum triglyceride levels in patients diagnosed with obesity and diabetes (Okanovic et al., 2015; Shay et al., 2009). Creatine kinase is an enzyme that promotes creatine phosphorylation and was accepted to be considered as an indirect marker of muscle damage, especially for the diagnosis of myocardial infarction or muscular dystrophy (Patel et al., 2013). In this study, the high level of creatine kinase suggested that muscle damage is due to IR in rats with HFD/STZ‐induced T2DM.

HFD causes excessive intercellular oxidative stress and stimulates proinflammatory cytokine secretion, resulting in the occurrence of IR and DM (Doody et al., 2017; Locksley et al., 2001). In addition, excessive cytokine production may inhibit muscle regeneration by regulating mTOR expression, which may subsequently lead to muscle loss in DM (Pelosi et al., 2014). ALA has been demonstrated to modulate nuclear factor kappa B, JNK, and PI3K/AKT signaling pathways in muscles (Hong et al., 2018; Rousseau et al., 2016). Pio is an anti‐inflammatory and antioxidant of diabetic rodents. A previous study suggested that the anti‐inflammatory and antioxidant abilities of Pio (10 mg/kg/day) are similar to those of ALA (100 mg/kg/day) in the peripheral nerves of diabetic rats (Jin et al., 2014). A similar result was observed in the muscle tissue in this study. Moreover, our previous study has also indicated that ALA improves pAKT/AKT ratios and showed a dose‐dependent manner (50–200 mg/kg/day) in liver tissues of diabetic rats (Ko, Lo, et al., 2021). In the present study, ALA ameliorated levels of TNF‐α and pAKT/AKT ratios in muscle tissues, suggesting that ALA may alleviate inflammation by suppressing the TNF‐α/JNK pathway in muscle of type 2 diabetic rats.

ALA enhanced the protein expression of PI3K/AKT in the soleus muscle and reduced IR in rats with T2DM in the present study. Our previous study also found that ALA enhanced PI3K/AKT protein expression in the liver and brain tissues of rats with T2DM (Ko, Lo, et al., 2021; Ko, Xu, et al., 2021). ALA increases IRS‐1 protein expression in muscles and increases the binding of IRS‐1 to regulatory subunit p85 of PI3K in obese Zucker rats (Shay et al., 2009). ALA also reduces Ser789 phosphorylation of IRS‐1 (Wozniak et al., 2012). However, no significant difference was observed in IRS‐1 protein expression in the muscles of rats with T2DM treated with ALA.

In general, low‐degree inflammation stimulates appropriate secretion of cytokines and subsequently stimulates insulin‐like growth factor (IGF)‐1 and fibroblast growth‐factor secretion for satellite cell proliferation. The increase in myogenic regulatory gene expression promotes the differentiation of satellite cells into myoblasts in the formation of muscle fibers (Karalaki et al., 2009), whereas excessive TNF‐α suppresses MyoD protein and inhibits muscle regeneration (Carotenuto et al., 2016). Conversely, PI3K/AKT‐pathway activation promotes the expression of the MyoD protein and promotes the differentiation of satellite cells, which constitute myoblasts. Similarly, PI3K/AKT‐pathway activation also promotes mTOR protein expression and muscle generation (Bodine et al., 2001; Xu & Wu, 2000). Normally, the IGF/PI3K/AKT pathway inhibits the performance of muscle‐decomposition‐related proteins by inhibiting FOXO protein expression. However, when FOXO protein expression increases, muscle atrophy is triggered (Foletta et al., 2011). In this study, MyoD and mTOR proteins were significantly increased in the ALA200 group, indicating that ALA promoted muscle regeneration and increased the muscle mass of T2DM rats. In addition, ALA also reduced the expressions of muscle‐degradation‐related proteins, such as FOXO and Murf1, which has not been previously reported.

MyHC is an indicator protein usually used to evaluate the lengths of muscle fibers and muscle mass. Studies suggest that MyHC may increase muscle regeneration and decrease muscle degradation to preserve muscle mass (Clarke et al., 2007; Muroya et al., 2002). ALA has been reported to promote the gene expression of MyHC and to preserve muscle mass in OLETF rats (Yoo et al., 2009). Our results are consistent with previous studies, suggesting that ALA may alleviate muscle degradation and promote muscle regeneration, consequently preserving muscle mass in T2DM rats.

## 5 CONCLUSIONS

In the present study, muscle loss was observed in rats with HFD/STZ‐induced T2DM. Treatment with ALA for 13 weeks significantly ameliorated soleus muscle atrophy and maintained the length of muscle fibers in T2DM rats. We postulate that ALA may alleviate inflammation by suppressing the TNF‐α/JNK pathway and may ameliorate insulin signal transduction by improving the PI3K/AKT pathway, thus increasing muscle generation and reducing muscle degradation, ultimately inhibiting atrophy in the muscle of T2DM rats.

## FUNDING INFORMATION

This study was funded by the Ministry of Science and Technology of the Republic of China (ROC; No. MOST 107‐2320‐B‐003‐004‐MY3) and the Second Affiliated Hospital of Fujian Medical University (serial No. BS201902).

## CONFLICT OF INTEREST

The authors declare that they do not have any conflict of interest.

## ETHICS STATEMENT

This study was conducted in accordance with the ethical guidelines of the Institutional Animal Care and Use Committee of National Taiwan Normal University; Taipei, Taiwan (approval no. 106042).

## Data Availability

Data used to support the findings of this study have been included in this article.
